# Pea‐based bulgur: a nutrient‐rich and high‐protein plant‐based food product

**DOI:** 10.1002/jsfa.14205

**Published:** 2025-03-01

**Authors:** Buket Cetiner

**Affiliations:** ^1^ Department of Quality and Technology Field Crops Central Research Institute Ankara Türkiye

**Keywords:** pea, bulgur, mineral bioavailability, high in protein, pulse, plant‐based

## Abstract

**BACKGROUND:**

Bulgur is a traditional food product and is widely consumed in Turkey and Middle Eastern countries. The main aim of this study was to investigate the potential of peas as a high‐protein plant‐based raw material in bulgur production.

**RESULTS:**

Cooked pea‐based bulgur produced from Deren and Irmak can provide 32.8% and 28.4% of its caloric content from protein. Therefore, ‘high in protein’ labeling can be used for both pea‐based bulgur samples. Bioavailabilities of Zn, Mg, P, Ca, Fe and Cu in the pea‐based bulgur (Deren) were determined as 44%, 29%, 17%, 10%, 16% and 31%, respectively. One serving portion of cooked pea‐based bulgur (Deren) could meet up to 28%, 26%, 15%, 9%, 44%, 15% and 51% of the daily mineral requirement for females of zinc, magnesium, phosphorus, calcium, manganese, iron and copper, respectively.

**CONCLUSION:**

The outcomes of this research are anticipated to provide an impetus for understanding the impact of pea utilization in bulgur production, providing insights that could inform the development of nutritious and sustainable plant‐based products to meet the demands of health‐conscious consumers. © 2025 The Author(s). *Journal of the Science of Food and Agriculture* published by John Wiley & Sons Ltd on behalf of Society of Chemical Industry.

## INTRODUCTION

Bulgur is one of the traditional foods commonly produced from wheat. It is widely consumed in Turkey and Middle Eastern countries. Bulgur is known for its quick cooking time and nutty taste, making it a popular and nutritious alternative to rice or couscous. Bulgur prepared from whole or crushed kernels by soaking in water, parboiling, drying and grinding has been a staple food for centuries.^1^ The first historical information on bulgur was found in the Catalhoyuk archaeological site in Anatolia (Turkey), which dates back 7000 to 8000 years.^2^ Bulgur is also referred to as bulgor, boulgur, burgul, broughoul, burghoul and burghul in different cultures.^3^

The global quest for sustainable and nutritionally rich food alternatives has spurred research into innovative approaches to staple grains. Pulses, which include dry peas, lentils, chickpeas and beans, are an essential component of a healthy and sustainable diet. The importance of pulses stems from their numerous nutritional, environmental and economic benefits. Pulses have long been important components of the human diet due to their content of starch, protein, fiber, essential vitamins and minerals.^4^, ^5^ They serve as an excellent plant‐based protein alternative for individuals, including vegetarians and vegans, contributing to overall health. Pulses are particularly noteworthy for their high protein content relative to other plant‐based foods. Additionally, they contain antioxidants that help combat oxidative stress in the body, potentially reducing the risk of chronic diseases.^4^, ^6^

Pea (*Pisum sativum* L.) is one of the world's main food legumes. *Pisum sativum* L. is one of the most common pulses that is economically important with a global annual production estimated at around 21 million tons, and it is grown today in around 90 countries The integration of peas into different products has significant importance due to their nutritional value and the cultivation of peas contributes to sustainable agricultural practices. Tulbek *et al*.^6^ have explored sustainable agricultural practices, emphasizing the environmental advantages associated with pea cultivation. Their ability to fix nitrogen reduces the environmental impact of farming by decreasing the reliance on nitrogen‐based fertilizers, which can lead to soil degradation and water pollution. Many studies have delved into the nutritional benefits of pea‐based products, highlighting their positive impact on dietary protein intake.^4^, ^5^, ^6^ Protein content in peas lies in the range of 21% to 30% with an average of 23% depending on genotype, growing environment and related factors such as temperature, rainfall and soil type.^7^, ^8^ The overall phenotypic expression of protein content is a result of environmental as well as genotypic components. Pea protein has garnered attention for its well‐balanced amino acid profile and has high nutritional value due to its relatively high content of lysine, an amino acid that limits the nutritional value of cereals.^8^ Pea proteins have a high potential to be utilized as ingredients in the food industry because of their relatively balanced amino acid profile compared with other plant proteins.^9^ Ensuring the delivery of nutrition to individuals while minimizing the environmental impact on ecosystems is crucial. Combining environmental and nutritional attributes into carbon and water footprints can be used to assess sustainable food options in the food sector. Life cycle assessment of cooked pea protein balls *versus* beef meatballs made from beef showed that pea protein balls have a smaller environmental impact across most impact categories assessed.^10^

Due to its richness in a variety of nutritional and bioactive ingredients, the consumption of pea has been suggested to be associated with a wide range of health benefits, and there has been increasing focus on its potential as a functional food.^5^, ^11^ The development of pea products has attracted much attention in the food industry.^11^ Minerals represent from 0.2% to 0.3% of the total intake of all nutrients in the diet. They are so potent and so important that without them an organism would not be able to utilize all the other food components. Minerals from food are required for the preservation of healthy tissue and cellular metabolism. Mineral absorption and biological availability are significantly influenced by the chemical form in which minerals are found in food. The rate and efficiency of mineral intake are determined by factors affecting their solubility, reduction to a state suitable for cellular uptake, transfer across the mucosa or transport into circulation.^12^ Bioavailability represents the fraction of a consumed compound or nutrient that reaches the systemic circulation and can be utilized. Nutrient bioavailability refers to the portion of food that the body absorbs and utilizes.^13^, ^14^ The bioavailability of minerals has become more of a focus with the increasing trend toward healthy food consumption. Pea is a good source of minerals including iron, zinc, calcium, potassium, magnesium, phosphorus and zinc.^15^

Several studies have investigated the potential of legumes as plant‐based raw material for bulgur production. Bilgiçli^16^ investigated the changes in physical and chemical properties of common bean and chickpea bulgur prepared with different cooking and drying processes. In another study, Yorgancilar and Bilgiçli^17^ used bitter and sweet lupin (*Lupinus albus* L.) seeds in bulgur production and stated that sweet lupin bulgur can be used as a new legume‐based product with good nutritional properties. Although considerable research has been undertaken to investigate the bulgur characteristics of durum wheat, bread wheat, barley and some pulses such as chickpea, bean and lupin,^1^, ^16^, ^17^, ^18^, ^19^, ^20^, ^21^, ^22^ we have not encountered any systematic studies investigating the potential of peas in bulgur production. This paper introduces a new approach to bulgur production using peas, aiming to elevate its nutritional profile beyond conventional standards. The overarching goal of the study reported here was to comprehensively investigate the potential of peas as a high‐protein raw material in bulgur production. The specific objectives were to (1) assess the physical properties and cooking characteristics of pea varieties, (2) evaluate the protein, ash contents, total phenolic content and antioxidant activity of peas and pea‐based bulgur samples, (3) evaluate the cooking properties, mineral content and mineral bioavailability of pea‐based bulgur samples, (4) examine the contribution of one portion of cooked pea‐based bulgur to the daily mineral intakes of females and males and (5) obtain bulgur which is compliant with international health claims about protein content.

## MATERIALS AND METHODS

### Materials

**Figure 1:**
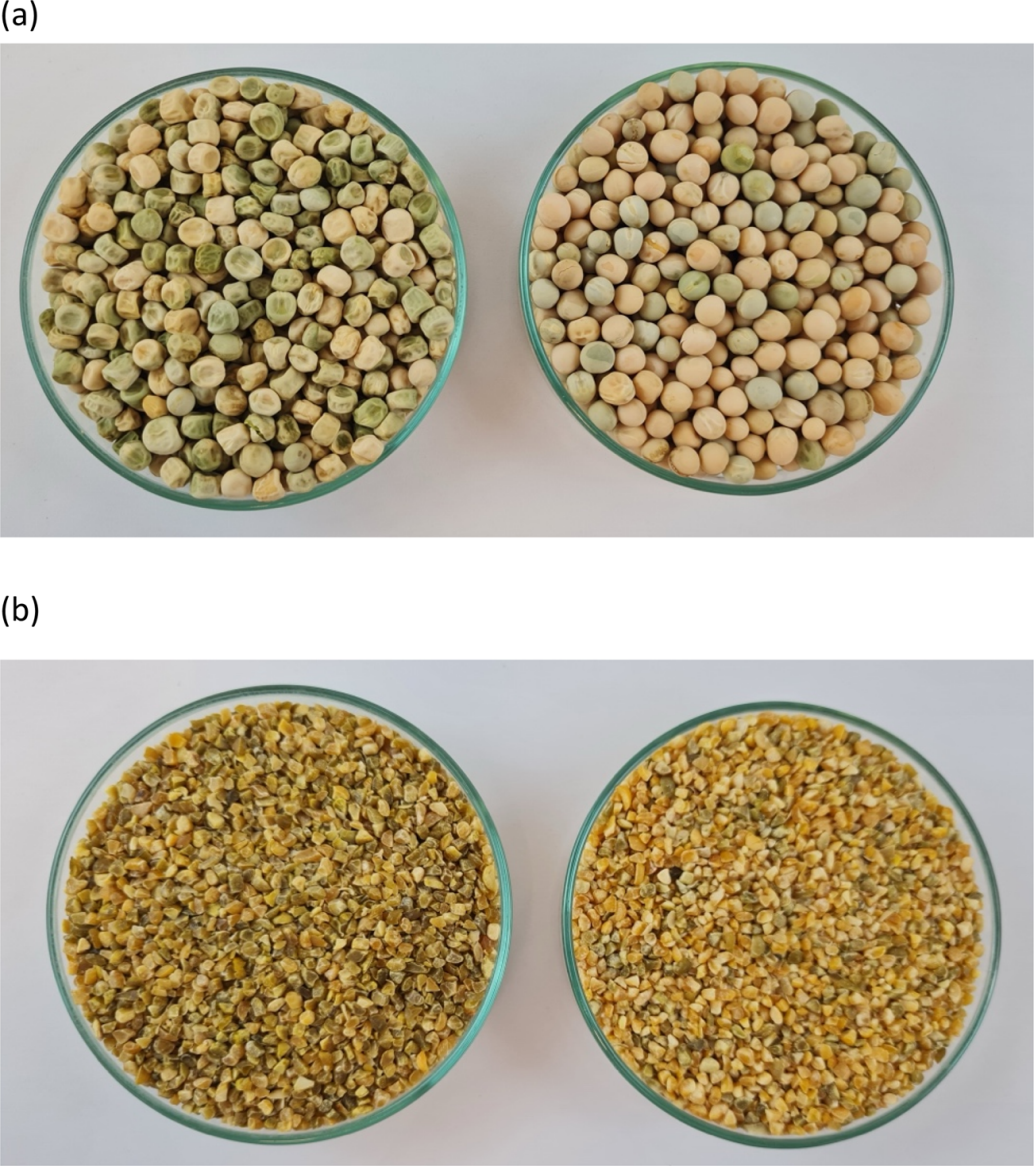
Photographs of (a) pea varieties (Deren and Irmak) and (b) their bulgur samples.

Pea (*Pisum sativum* L.) varieties Deren and Irmak‐01 were used in the study. They were obtained from the Eastern Mediterranean Agricultural Research Institute (Adana, Turkey). Pea varieties were grown in the Adana location in Turkey in the 2021–2022 growing season. Deren had rough or wrinkled seeds and Irmak had smooth or rounded seeds (Fig. 1). The name of the variety Irmak‐01 is shortened as Irmak in the text for convenience.

#### Milling

Wholemeal samples were obtained using a ZM200 mill (500 μm sieve, Retsch Haan, Germany). Each bulgur sample was ground using a mixer with a ceramic blade (Buchi Mixer B‐400, Flawil, Switzerland) and sieved through a 500 μm screen.

### Methods

#### Bulgur production

Cooking: Bulgur samples were produced by cooking under atmospheric pressure. The cleaned pea sample was cooked with tap water (100 °C) and cooking was performed until the starch in the endosperm was entirely gelatinized. The amount of water used in cooking pea bulgur was determined by preliminary studies, resulting in a pea‐to‐water ratio of 1.0:1.5 (w/v).

Cooked samples were kept at room temperature for 30 min to vaporize surface moisture before drying. Drying, tempering, debranning and cracking procedures were done according to the method described in Kaplan and Özkaya.^19^

Drying: Samples were placed over trays and dried in a drying cabinet (Protech TT107, Ankara, Turkey) at 50 °C until the water content of the sample decreased to between 9% and 11%.^19^

Tempering, debranning and cracking: Tempering was used on dried samples to remove easily the seed coat from the seed's surface. Before debranning, the samples were tempered for increasing the moisture content by 2% for 30 min. A debranner (Poyraz Muhendislik, Konya, Turkey) was used for 6 min to remove the seed coat of the pea sample. A hammer mill (Duru Degirmen Makinalar, Karaman, Turkey) was used to crack the debranned pea samples and samples were sieved through a 0.5 mm sieve to remove fine particles. The bulgur samples were kept in a refrigerator at 4 °C until further analyses. Bulgur yield was calculated by dividing the final bulgur amount by initial seed amount.^19^

#### Characterization of pea and bulgur samples

The protein content of the pea and bulgur samples was assessed through combustion nitrogen analysis (Leco FP828, St Joseph, MI, USA) calibrated with EDTA following AACCI method 46‐30.^23^ The correction factor was used as 6.25 for pea and pea bulgur samples. Moisture and ash content of the pea and bulgur samples were determined using AACCI methods 44‐15A and 08‐01.01,^23^ respectively. The color values (L*, a*, b*) of pea and bulgur samples, under D65 illuminant and 10° observer conditions, were determined using a spectrophotometer (Miniscan, HunterLab, Reston, VA, USA) following method E 1164.^24^

#### Soaking and cooking properties of pea samples

The soaking properties determined for pea samples were dry and wet seed weights, dry and wet seed volumes, water absorption capacity, water absorption index, swelling capacity and swelling index. The cooking properties determined for the pea samples were cooking time according to the modified methods of Williams *et al*.^25^

Kernels (100) were weighed to get dry seed weight (g) and they were placed into a cylinder. Water was added into the cylinder and the added amount of water was subtracted from the read volume value from the cylinder to get dry seed volume. Kernels (100) were soaked with water (*ca* 200 mL) in a beaker for 16 h. The kernels were removed from the water, dried with drying paper and weighed to determine their wet seed weight (g). They were placed into cylinders. Distilled water was added into the cylinder and the added value was subtracted from the recorded value to get wet seed volume. These soaked (wet) pea kernel samples were used for cooking time determination.

(1) $$\text{Water absorption capacity}=\begin{array}{l} \frac{\mathrm{wet}\;\text{seed weight after soaking}–\mathrm{dry}\;\text{seed weight before soaking}}{100-\text{number of nonswelling seeds}}\\ \times 100 \end{array}$$

(2) $$\text{Water absorption index}\;\left(\%\right)=\frac{\;\text{water absorption capacity}}{\mathrm{dry}\;\text{seed weight before soaking}}\times 100$$

(3) $$S\text{welling capacity}=\frac{\mathrm{wet}\;\text{seed volume after soaking}–\mathrm{dry}\;\text{seed volume before soaking}}{100-\text{number of nonswelling seeds}}$$

(4) $$\text{Swelling index}=\frac{\text{swelling capacity}}{\mathrm{dry}\;\text{seed volume before soaking}\;}\times 100$$

A crude fiber apparatus (Labconco, Kansas City, MO, USA) was used for the cooking time determination of pea samples. Beakers were used for boiling the samples under continuous reflux. Cooking time was determined by calculating the time between the starting boiling time and the time at which kernels are ready to eat. Kernel cooking indicates the gelatinization of the starch and simultaneous reduction of the cell wall tissues to the point where they are soft and friable enough to break into pieces easily in the mouth.^25^

#### Cooking properties of pea bulgur samples

The cooking properties determined for the bulgur samples were cooking time, weight and volume increase during cooking, and cooking loss. The cooking process was carried out by using a crude fiber apparatus (Labconco, Kansas City, MO, USA). Beakers were used for boiling the samples under continuous reflux. For the determination of cooking time, 10 g of bulgur sample and 100 mL of water were used. Cooking time was determined by calculating the time between starting boiling time and the time at which kernels are ready to eat. Cooking was continued until there was no hard/opaque uncooked part in the center of the bulgur particles. After cooking, the sample was removed from the water, dried with drying paper and weighed to determine cooked bulgur weight (g) and water absorption was calculated according to Eqn (5):

(5) $$\text{Bulgur water absorption}\left(\%\right)=\begin{array}{l} \frac{\text{cooked bulgur weight}-\text{initial bulgur weight}}{\text{initial bulgur weight}}\\ \times 100 \end{array}$$

To determine the volume increase, 10 g of bulgur was placed into a measuring cylinder containing a certain amount of water. The increase in volume is the dry bulgur volume. After cooking the bulgur, the sample was removed from the water, dried with drying paper and placed into a measuring cylinder containing a certain amount of water. The increase in water level is the volume of cooked bulgur. The volume increase was calculated according to Eqn (6):

(6) $$\text{Volume increase}\left(\%\right)=\begin{array}{l} \frac{\text{cooked bulgur volume}-\mathrm{dry}\;\text{bulgur volume}}{\mathrm{dry}\;\text{bulgur volume}}\\ \times 100 \end{array}$$

Cooking loss quantifies the solid content leached from the bulgur into the cooking water. The cooking water, gathered in a pre‐weighed beaker, underwent evaporation at 100 ± 1 °C in an oven. The resulting residue was weighed and documented as a percentage of the initial material following the modified procedure outlined in AACCI method 66‐50.01.^23^

#### Total phenolic content and antioxidant activity measurements

A ground bulgur sample underwent sieving using a 500 μm sieve. The sample (0.25 g) was blended with a 10 mL solution of methanol and distilled water (50:50). Subsequently, the mixture underwent incubation with shaking at 250 rpm at room temperature for 12 h in the dark. Following the incubation period, the mixture was centrifuged at 2800 × *g* for 10 min using a centrifuge (Sigma, Osterode am Harz, Germany). The resulting supernatant was filtered through Whatman No. 1 filter paper.

The determination of total phenolic content (TPC) in the samples was carried out spectrophotometrically, following a modified method as described by Singleton and Rossi.^26^ Briefly, each of the pea and bulgur extracts (0.1 mL) was mixed with 0.5 mL of Folin–Ciocalteu phenol reagent. Then, 1.5 mL of Na_2_CO_3_ solution (20%, prepared with distilled water) was added to this mixture and the total volume was completed to 10 mL by adding distilled water. Following 120 min of incubation in the dark at room temperature, the absorbance value was measured at 765 nm using a UV–visible spectrophotometer (Hitachi U1800, Tokyo, Japan). A calibration curve was generated using gallic acid as a standard. The TPC of the samples was quantified and expressed as milligrams of gallic acid equivalent (GAE) per 100 g of the sample (mg GAE (100 g)^−1^).

The 1,1‐diphenyl‐2‐picrylhydrazyl (DPPH) radical scavenging activity of the pea and pea‐based bulgur extracts was determined following the modified method described by Singh *et al*.^27^ An amount of 100 μL of the extract was combined with 3.9 mL of freshly prepared DPPH radical solution (3.9 mg of DPPH in 100 mL of methanol). After a 120 min incubation in the dark at room temperature, the absorbance value was measured at 515 nm using a spectrophotometer (Hitachi U1800, Tokyo, Japan). The results were expressed as milligrams of Trolox equivalent per 100 g of the sample (mg TE (100 g)^−1^).

#### Mineral content determination and mineral bioavailability

Samples were digested with 5 mL of HNO_3_ (65%) and 2 mL of H_2_O_2_ (30%) in a microwave system (Mars 6; CEM Corp., Matthews, NC, USA). After digestion, ultrapure water (Milli‐Q Ultrapure Water System, Merck Millipore, MA, USA) was added to complete the total volume to 20 mL. Digestates were kept in a refrigerator (+4 °C) until the analysis. The mineral content of digestates was determined using inductively coupled plasma mass spectrometry (Agilent 7850, Agilent Technologies, Wilmington, DE, USA). The results were recorded as ppm or ppb.

Enzymatic digestion of bulgur samples was performed according to Suliburska and Krejpcio^28^ for *in vitro* mineral bioavailability analysis. The method is described in detailed in Cetiner *et al*.^13^

#### Statistical analysis

All experiments were performed in duplicate and the mean values and standard deviations were calculated using Excel (Microsoft Office Professional Plus 2016). Results were reported as mean ± standard deviation. Some results were analyzed using a one‐way analysis of variance and statistical analysis was performed with JMP software (version 11.0.0, SAS Institute Inc., 2013). When significant (*P* < 0.05) differences were found, the least significant difference was used to determine the differences among means.

## RESULTS AND DISCUSSION

### Quality evaluation of pea and bulgur samples

The hundred‐seed weight, hectoliter weight and seed size distribution of the pea varieties are presented in Table 1. The hundred‐seed weights of Deren and Irmak were 13.3 and 16.9 g while hectoliter weights were 74.6 and 80.0 kg hL^−1^, respectively. Santos *et al*.^29^ analyzed a set of pea accessions and stated that their hundred‐seed weights were in the range of 5–29 g with a mean of 18 g. The seed size measurement showed that there were similar trends in distribution for both varieties (Deren and Irmak). More than 55% of both samples had a seed size between 6 and 7 mm. The results of the present study are in line with the results of Shahin and Symons^30^ who measured the seed size of different pulses with image analysis systems and stated that the size of green pea samples was mainly distributed in the range of 5.95 and 7.14 mm.

**Table 1:** Physical, soaking and cooking properties of pea varieties Deren and Irmak

Pea	Physical properties						Soaking and cooking properties					
	Hundred weight (dry seed weight) (g, dwb)	Hectoliter weight (kg hL^−1^)	Sieve analysis				Water absorption (g H_2_O/seed)	Water absorption capacity (g H_2_O kg^−1^ seed)	Water absorption index (%)	Swelling capacity (mL/kernel)	Swelling index (%)	Pea cooking time (min)
			≥8 mm	8 > *x* (mm) ≥ 7	7 > *x* (mm) ≥ 6	Under sieve (<6 mm)						
Deren	13.3 ± 0.3	74.6 ± 0.9	0 ± 0	16.42 ± 0.72	57.42 ± 0.98	26.16 ± 1.70	0.207 ± 0.002	1556 ± 12	1.357 ± 0.042	0.202 ± 0.002	2.57 ± 0.04	50 ± 4
Irmak	16.9 ± 0.5	80.0 ± 0.1	0 ± 0	13.47 ± 2.74	55.37 ± 2.55	31.17 ± 5.28	0.190 ± 0.003	1121 ± 16	0.999 ± 0.031	0.190 ± 0.000	2.31 ± 0.06	49 ± 13

Values are presented as mean ± standard deviation.

dwb, dry weight basis.

Cooking of pulses is a hydrothermal process that involves starch gelatinization, protein denaturation and structural softening.^31^ The water absorption (hydration) capacities of the samples were 1556 and 1121 g H_2_O kg^−1^ seed for Deren and Irmak, respectively. The values were higher than those of Wang *et al*.^31^ who determined the hydration capacity of yellow pea samples in the range of 855–979 g H_2_O kg^−1^ seed. Wang *et al*.^32^ reported results very similar to those of the present study and concluded that the water absorption (hydration) capacities of 120 pea samples were in the wide range of 936–1316.6 g H_2_O kg^−1^ seed. Water absorption (hydration) capacities of the bulgur samples were affected by species/variety, cooking method, grain structure and particle size.^20^

The protein and ash contents of the pea and bulgur samples are given in Table 2. The protein contents of Deren and Irmak were 29.4% and 26.2%, while their ash contents were 3.56% and 2.82%, respectively. The ash content results were in line with the study of Abdel‐Aal *et al*.^33^ They stated that ash contents of pea samples were in the range of 3.3–3.7%. Seed surface shape has been reported to be related to pea protein content. Wrinkled‐seeded types have higher protein and lower starch content relative to the round‐seeded types.^29^, ^34^ In the present study, the wrinkled‐shaped cultivar Deren had a higher protein content compared to the smooth and round‐shaped cultivar Irmak.

**Table 2:** Chemical properties and color values of pea and bulgur samples

Sample		Chemical properties		Color values		
		Protein (dwb, %)	Ash (dwb, %)	L*	a*	b*
Pea	Deren	29.4 ± 0.3^b^	3.56 ± 0.01^a^	57.14 ± 0.60^b^	1.61 ± 0.26^d^	22.01 ± 0.08^c^
Pea – Irmak		26.2 ± 0.4^c^	2.82 ± 0.01^bc^	61.76 ± 1.65^a^	5.00 ± 0.72^c^	21.64 ± 0.31^c^
Bulgur	Deren	33.4 ± 0.1^a^	3.16 ± 0.16^b^	52.78 ± 0.74^c^	6.84 ± 0.06^b^	29.83 ± 0.35^b^
Bulgur – Irmak		29.0 ± 0.1^b^	2.51 ± 0.16^c^	58.45 ± 0.42^b^	9.50 ± 0.13^a^	33.29 ± 0.11^a^

Values followed by different letters in the same column are significantly different (*P* < 0.05). Values are presented as mean ± standard deviation.

dwb, dry weight basis.

Bulgur was produced from the pea cultivars Deren and Irmak with yields of 80.6% and 79.2%, respectively (Table 3). It was calculated as the ratio of the final bulgur weight to the initial seed weight. Bulgur yield, an essential parameter for producers, is influenced by factors such as variety/species, as well as cooking, drying and milling conditions.^18^ The ash contents of the bulgur samples (Deren and Irmak) were 3.16% and 2.51%, respectively (Table 2). Ash is the inorganic residue and serves as an indicator of mineral levels in legume products; therefore, a high ash content is an important indicator of the mineral content.^33^ In the study of Köksel *et al*.,^1^ they used three barley cultivars and processed them into bulgur by two different cooking methods: pressure cooking or cooking at atmospheric pressure. They investigated the effect of processing on different parameters and they reported that the ash content of the samples decreased from grain to bulgur for both cooking methods. These results are in line with those of the present study in that the ash contents decreased from pea to bulgur for both pea varieties.

**Table 3:** Bulgur yield and cooking properties of bulgur samples

Sample	Bulgur yield (%)	Cooking properties of bulgur			
		Cooking time (min)	Water absorption (%)	Volume increase (%)	Cooking loss (%)
Deren	80.6 ± 2.1	17.3 ± 1.1	181.6 ± 10.9	221.9 ± 4.4	17.9 ± 1.8
Irmak	79.2 ± 0.6	18.3 ± 1.1	113.2 ± 14.2	102.4 ± 10.8	38.7 ± 5.4

Values are presented as mean ± standard deviation.

Pea (*Pisum sativum* L.) is an important source of high‐quality vegetable protein in the human diet and consuming pea is associated with many health benefits.^11^ The protein contents of the bulgur samples prepared from Deren and Irmak were 33.4% and 29.0%, respectively (Table 2). The protein content of pea bulgur samples was higher than that of the respective grains. During bulgur production, cooked and dried pea samples were debranned. The reason for the increase in protein content may be due to the removal of the hull of pea samples during bulgur production. The protein content is different in the hull, cotyledon and germ parts of the pea seed. Kosson *et al*.^35^ studied the protein and lipid distribution of cotyledon, hull and germ layers of three pea cultivars and concluded that the protein contents of hull part were quite low (in the range of 3.5–4.4%) while those of whole seeds were in the range of 19.1–30.5%.

The serving size of cooked bulgur is about 120 g per portion.^36^ The amount of pea bulgur required to obtain one serving of cooked pea bulgur was calculated using the bulgur cooking analysis results. This information was used together with the pea bulgur protein analysis results to calculate the amount of protein in one serving of cooked pea bulgur. The amount of uncooked pea bulgur required to obtain one serving of cooked pea bulgur (120 g) was calculated as 38.2 and 50.7 g, and the amount of protein in one serving cooked of pea bulgur were calculated as 12.7 and 14.6 g for Deren and Irmak, respectively. Regarding the claim on the label of food related to protein, there are two options, ‘source of protein’ and ‘high protein’, described in Regulation (EC) No. 1924/2006 of the European Parliament and of the Council on Nutrition and Health Claims Made on Foods.^37^ A claim that a food is a source of protein may only be made where at least 12% of the energy value of the food is provided by protein while a claim that a food is high in protein may only be made where at least 20% of the energy value of the food is provided by protein. According to the Guidelines on Nutrition Labelling,^38^ the amount of energy was calculated by using the following conversion factors: carbohydrates, 4 kcal g^−1^; protein, 4 kcal g^−1^; fat, 9 kcal g^−1^. The fat content of the peas was found to be quite low, ranging between 0.0067% and 0.0169%, as reported in several studies.^32^, ^39^, ^40^ For energy calculations, it was assumed to be 1%. The results of the present study indicated that it is possible to get 32.8 ± 0.3% and 28.4 ± 0.0% of calories from protein per serving of cooked pea bulgur produced from the high‐protein pea varieties Deren and Irmak. It can be clearly seen that more than 20% of calories are taken from protein per serving of cooked pea bulgur produced from Deren and Irmak. Therefore, the nutritional claim ‘high in protein’ can be used in labeling for both Deren and Irmak pea bulgurs according to Regulation (EC) No. 1924/2006.^37^ Compared to other plant proteins, pea protein is characterized by its high digestibility, relatively low allergenic responses and fewer negative health controversies.^41^, ^42^, ^43^

### TPC and antioxidant activity of pea and bulgur samples

The TPC and antioxidant activity results of the pea and bulgur samples are presented in Table 4. The TPCs of Deren and Irmak were found as 101.3 and 104.2 mg GAE (100 g)^−1^, while the TPCs of pea bulgur samples produced using Deren and Irmak were lower at 81.4 and 90.3 mg GAE (100 g)^−1^, respectively. The difference between TPC values of kernel and bulgur was significant for only the Deren variety. During bulgur production, TPC levels decreased for both samples. In Deren and Irmak samples, the TPC losses during bulgur production were determined to be approximately 20% and 13%, respectively. The DPPH radical scavenging capacity changed from 70.1 to 63.7 mg TE (100 g)^−1^ for Deren and from 96.6 to 87.2 mg TE (100 g)^−1^ for Irmak as the DPPH values of grain and bulgur samples were compared. Tekin‐Cakmak *et al*.^20^ investigated the TPC and antioxidant activity of barley and durum wheat samples and bulgur samples produced from them. Similar to the present study, TPC and DPPH antioxidant activity values of the kernel samples were higher than those of the respective bulgur samples. Oomah *et al*.^44^ investigated the phenolic content and antioxidant activity of whole grain, hull and residue of green lentil, red lentil and yellow pea. They reported that the phenolic content and antioxidant activity were higher in the hulls of the legumes. In the current study, during bulgur production, cooked and dried pea samples were debranned. The observed decrease in TPC and antioxidant activity may be attributed to the removal of the hulls from the pea samples during the debranning process.

**Table 4:** Phenolic content and antioxidant activity of bulgur samples

Sample	Total phenolic content (mg GAE (100 g)^−1^)	DPPH antioxidant activity (mg TE (100 g)^−1^)
Deren – grain	101.3 ± 2.4^a^	70.1 ± 1.5^a^
Irmak – grain	104.2 ± 2.4^a^	96.6 ± 13.4^a^
Deren – bulgur	81.4 ± 9.7^b^	63.7 ± 20.8^a^
Irmak – bulgur	90.3 ± 0.0^ab^	87.2 ± 13.3^a^

Values followed by different letters in the same column are significantly different (*P* < 0.05). Values are presented as mean ± standard deviation.

GA, gallic acid equivalent; DPPH, 1,1‐diphenyl‐2‐picrylhydrazyl; TE, Trolox equivalent.

### Mineral content and mineral bioavailability of pea bulgur samples

Mineral contents (Zn, Mg, P, Ca, Mn, Fe and Cu) of the bulgur samples are given in Table 5. Mineral contents may vary depending on variety, location, soil, climate, environmental factors such as temperature, use of fertilizers, the state of the plant maturity at harvest and the interaction of all these factors.^45^, ^46^ The cultivars used in the study were grown in the same location under identical conditions and agricultural practices. Consequently, differences in mineral content among cultivars could be primarily attributed to the differences of the cultivar.

**Table 5:** Mineral content and mineral bioavailability of bulgur samples

Sample (bulgur)	Mineral content						
	Zn (ppm)	Mg (ppm)	P (ppm)	Ca (ppm)	Mn (ppm)	Fe (ppm)	Cu (ppm)
Deren	52.7 ± 4.7	1857 ± 240	2516 ± 4	2081 ± 216	18.3 ± 1.7	63.6 ± 6.8	10.7 ± 1.2
Irmak	40.0 ± 4.0	1509 ± 209	2561 ± 12	1037 ± 108	14.6 ± 1.5	59.9 ± 6.6	9.8 ± 1.2

Sample (bulgur)	Mineral bioavailability					
	Zn (%)	Mg (%)	P (%)	Ca (%)	Fe (%)	Cu (%)
Deren	44 ± 2	29 ± 2	17 ± 0.4	10 ± 0.1	16 ± 1	31 ± 3
Irmak	48 ± 4	43 ± 5	18 ± 0.1	33 ± 3	37 ± 3	16 ± 2

Values are presented as mean ± standard deviation.

The Zn, Mg, P, Ca, Mn, Fe and Cu contents (ppm) of Deren and Irmak were 52.7 and 40.0; 1857 and 1509; 2516 and 2561; 2081 and 1037; 18.3 and 14.6; 63.6 and 59.9; 10.7 and 9.8, respectively. Rasheed *et al*.^47^ studied the nutritional properties of bulgur prepared from wheat cultivars and concluded that the mineral composition of bulgur revealed that calcium and iron contents ranged from 368 to 532 and from 30 to 53 mg kg^−1^, respectively. Tekin‐Cakmak *et al*.^20^ examined the mineral composition of the bulgur produced using barley and durum wheat samples. They stated that Mg, Ca, Mn, Fe, Cu and Zn contents were in the range of 1016–1463, 82–112, 18.7–33.9, 28.7–61.8, 5.08–7.30 and 19.7–31.1 ppm, respectively. The results of the present study indicated that the Mg, Ca, Fe, Cu and Zn contents of pea bulgur were of comparatively higher values as compared to wheat bulgur and barley bulgur.

The portion of a nutrient that is available for the body to use is known as bioavailability, and it is a crucial nutritional feature.^14^, ^48^, ^49^ By simulating gastrointestinal digestion and measuring the mineral content in soluble fraction, it is possible to estimate the *in vitro* bioavailability of minerals in food products.^50^ The ratio of the solubilized mineral content following digestion to the original mineral content in pea bulgur was used to calculate the mineral bioavailability. Mineral bioavailability values of pea bulgur samples produced using Deren and Irmak are given in Table 5. Despite the high mineral content of peas, bioavailability may be lower due to phytate concentration. Sandberg^51^ stated that phytate acts as an inhibitor of Zn, Fe and Ca absorption; however, Trinidad *et al*.^52^ concluded that pulses’ phytate content had an effect on Fe bioavailability but it did not have an effect on Zn and Ca bioavailability.^4^ On the other hand, the bulgur production process has an effect of decreasing the phytic acid content.^18^ Kaplan and Özkaya^19^ investigated the effects of different wheat cultivars, cooking methods and bulgur types on the phytic acid content of bulgur. It was reported that the bulgur production process resulted in a decrease in phytic acid content with a mean of 37.7% from grain to bulgur. Cereal‐based foods such as bulgur represent a significant part of the diet of many countries. Hence, improving bulgar health constituents will be an advantage.^20^ The high protein content and mineral bioavailability observed in pea‐based bulgur not only support its classification as a ‘high in protein’ product but also highlight its potential to address nutritional deficiencies in plant‐based diets. These findings align with the study's objective to evaluate the nutritional quality of pea‐based bulgur and suggest its broader applicability as a nutrient‐rich alternative in the global market for plant‐based foods.

Daily mineral requirements for females and males based on intake recommendations were defined by Raymond and Morrow.^53^ The contributions to mineral intake by consuming one serving (120 g) of cooked pea bulgur to the daily mineral requirement of females and males were calculated by using the data of water absorption value (%) and mineral contents of bulgur samples and daily mineral intake recommendations. The results are given in Table 6. According to the data in Table 6, one serving (120 g) of cooked Irmak bulgur could meet up to 29%, 28%, 21%, 6%, 47%, 19% and 63% of the daily mineral requirements for females of zinc, magnesium, phosphorus, calcium, manganese, iron and copper, respectively. The finding is significant as it indicates that cooked pea‐based bulgur may become an important natural source of easily accessible minerals for consumers. Further research is needed to confirm the nutritional benefits of bulgur produced with peas and other pulses.

**Table 6:** Contribution of 120 g of cooked pea bulgur to mineral intakes and daily mineral requirement^a^

	Zn (mg d^−1^)	Mg (mg d^−1^)	P (mg d^−1^)	Ca (mg d^−1^)	Mn (mg d^−1^)	Fe (mg d^−1^)	Cu (μg d^−1^)
Intake recommendation	F: 8 M: 11	F: 310 M: 400	F: 700 M: 700	F: 1000 M: 1000	F: 1.8 M: 2.3	F: 18 M: 8	F: 900 M: 900
Bulgur sample	Percentage of 120 g of cooked pea bulgur meeting daily mineral requirement (%)						
Deren	F: 28 ± 1 M: 21 ± 1	F: 26 ± 1 M: 20 ± 1	F: 15 ± 1 M: 15 ± 1	F: 9 ± 0 M: 9 ± 0	F: 44 ± 2 M: 34 ± 2	F: 15 ± 1 M: 34 ± 2	F: 51 ± 2 M: 51 ± 2
Irmak	F: 29 ± 2 M: 21 ± 1	F: 28 ± 2 M: 22 ± 1	F: 21 ± 2 M: 21 ± 2	F: 6 ± 0 M: 6 ± 0	F: 47 ± 3 M: 37 ± 3	F: 19 ± 1 M: 43 ± 3	F: 63 ± 5 M: 63 ± 5

^a^ F and M: healthy adult between 19 and 30 years of age, female and male.

Values are presented as mean ± standard deviation.

## CONCLUSION

Nutrition stands at the forefront of optimizing human health, and dietary choices play a pivotal role in achieving this goal. In recent years, there has been a growing emphasis on the importance of incorporating high‐protein foods into diets, recognizing the indispensable role of proteins in fundamental biological processes. Bulgur, a globally consumed grain product, traditionally processed from wheat, is a staple in many diets. However, this study introduces a new approach to bulgur production using peas, aiming to elevate its nutritional profile beyond conventional standards. By using peas in the traditional bulgur formulation, it was aimed to improve its protein content and also tap into the nutritional benefits offered by peas, such as minerals, phenolics and antioxidants. Cooked pea‐based bulgur provided 28.4–32.8% of its caloric content from protein qualifying it for ‘high in protein’ labeling. The bioavailability of minerals in pea‐based bulgur was determined as follows: zinc (44–48%), magnesium (29–43%), phosphorus (17–18%), calcium (10–33%), iron (16–37%) and copper (16–31%). Additionally, a single serving of cooked pea‐based bulgur can contribute up to 28–29%, 26–28%, 15–21%, 6–9%, 44–47%, 15–19% and 51–63% of the daily mineral requirements for females of zinc, magnesium, phosphorus, calcium, manganese, iron and copper, respectively. The integration of peas into bulgur as a novel product is promising, offering a viable alternative to traditional products and aligning with the current emphasis on sustainable and health‐conscious food choices.

This study contributes to the scientific understanding of novel products and offers practical insights for both the food industry and consumers seeking diverse, nutritionally enriched options. Further studies should be carried out to meet the increasing demand for plant‐based products.

## CONFLICT OF INTEREST

The author declares no competing interests.

## Data Availability

Data will be made available on request.
